# A Genetic Code Expansion‐Derived Molecular Beacon for the Detection of Intracellular Amyloid‐β Peptide Generation

**DOI:** 10.1002/ange.202010703

**Published:** 2020-12-15

**Authors:** Khomkrit Sappakhaw, Krittapas Jantarug, Sarah A. Slavoff, Nipan Israsena, Chayasith Uttamapinant

**Affiliations:** ^1^ School of Biomolecular Science and Engineering Vidyasirimedhi Institute of Science and Technology (VISTEC) Rayong 21210 Thailand; ^2^ Department of Chemistry Yale University New Haven CT 06520 USA; ^3^ Stem Cell and Cell Therapy Research Unit and Department of Pharmacology Faculty of Medicine Chulalongkorn University Bangkok 10330 Thailand

**Keywords:** amyloids, genetic code expansion, proteoforms, proteolytic processing

## Abstract

Polypeptides generated from proteolytic processing of protein precursors, or proteolytic proteoforms, play an important role in diverse biological functions and diseases. However, their often‐small size and intricate post‐translational biogenesis preclude the use of simple genetic tagging in their cellular studies. Herein, we develop a labeling strategy for this class of proteoforms, based on residue‐specific genetic code expansion labeling with a molecular beacon design. We demonstrate the utility of such a design by creating a molecular beacon reporter to detect amyloid‐β peptides, known to be involved in the pathogenesis of Alzheimer's disease, as they are produced from amyloid precursor protein (APP) along the endocytic pathway of living cells.

## Graphical abstract

Biogenesis of amyloid‐β peptides from their precursor protein can be tracked in endocytic compartments of living cells by a molecular beacon labeling strategy based on genetic code expansion.

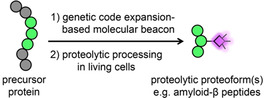

Proteolytic processing generates protein isoforms‐or proteoforms‐with function and properties distinct from their precursors. These proteolytic proteoforms regulate various biological processes and act as hormones, neurotransmitters, and disease pathogens.^[1]^ At the systems level, some proteolytic proteoforms can be discovered by proteomic techniques that enrich for the N‐terminus of polypeptide fragments.^[2]^ Targeted proteomic techniques can then be used to validate *bona fide* generation of these proteoforms. In cells and in vivo, proteolytic processing events can be investigated using protease reporter substrates and activity‐based probes.^[3]^ However, much less is known about the spatiotemporal dynamics of proteolytic proteoforms themselves. These proteoforms are often short peptides, and due to their small size and intricate site‐specific proteolytic processing, it is not trivial to append a genetic tag‐even a peptide epitope‐to these peptides for cellular studies while maintaining their proper biogenesis.

Genetic code expansion via the use of an orthogonal aminoacyl‐tRNA synthetase/tRNA pair has emerged as a powerful tool to label proteins in living mammalian cells.^[4]^ One approach employs a two‐step labeling strategy to affect biophysical probe attachment onto polypeptides (Figure 1 a). First, an amino acid bearing a bioorthogonal functional handle is co‐translationally inserted into a protein/peptide of interest. The functional handle is then chemically derivatized with a probe of choice. With its single‐amino acid label, genetic code expansion can be used to precisely place a biophysical tag within small proteins or peptides with minimal functional perturbation, and has been used to construct conformational sensors^[5]^ and to provide insight into functional dynamics of microproteins.^[6]^ Despite the ability to precisely place the biophysical label, genetic code expansion by itself does not allow selective visualization of the proteolytic proteoforms in the presence of excess of their precursors, the latter of which also carry the same label.

**Figure 1:**
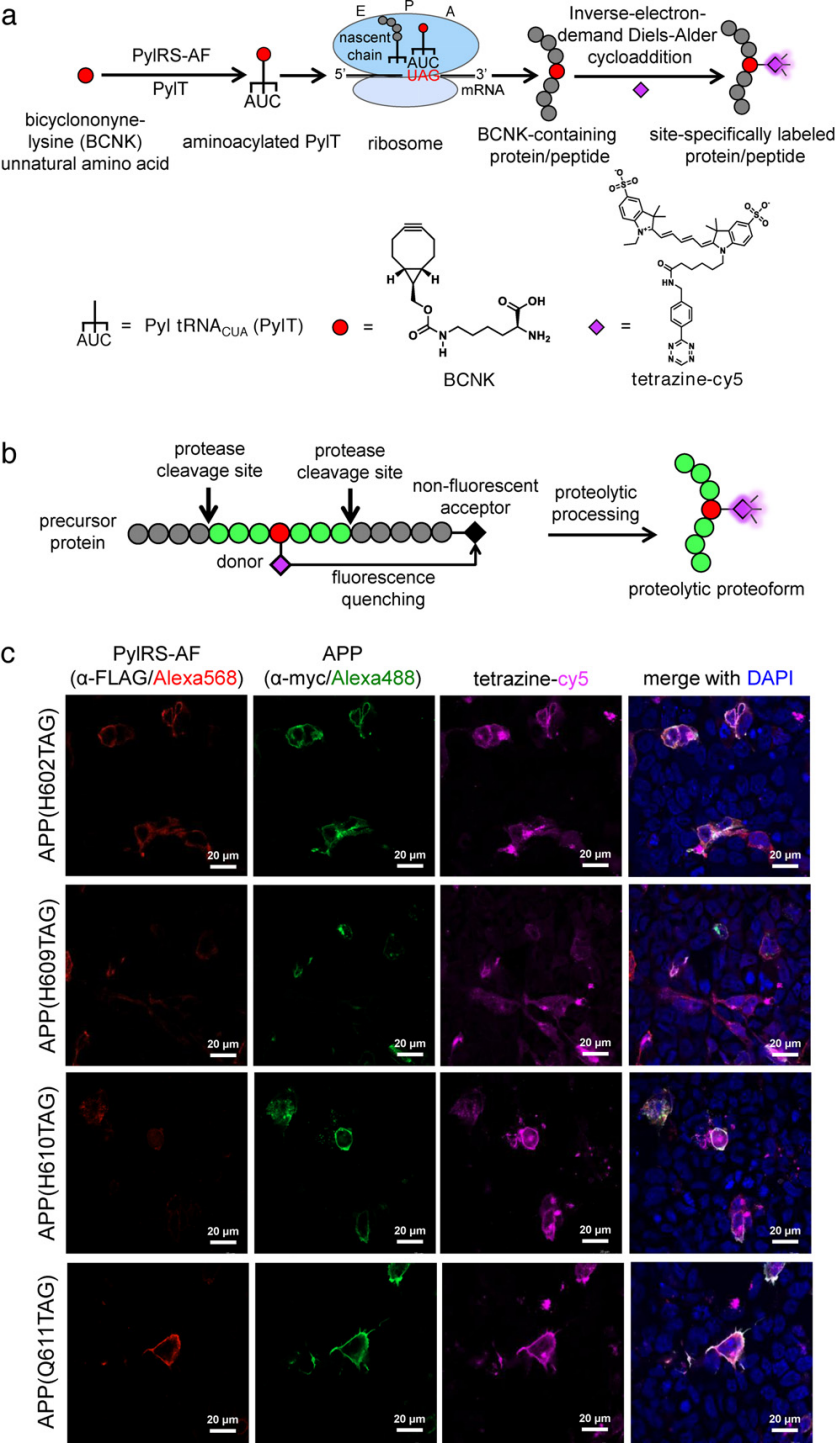
Genetic code expansion‐derived molecular beacon to detect proteolytic proteoforms. (a) Protein labeling by genetic code expansion. An unnatural amino acid, bicyclo[6.1.0]nonyne‐lysine (BCNK, red circle), is recognized by an orthogonal pyrrolysyl‐tRNA synthetase bearing Y306A/Y384F mutations (PylRS‐AF) and aminoacylated onto an amber‐suppressor pyrrolysyl tRNA (PylT). The BCNK‐charged PylT is decoded on the ribosome in response to the amber codon introduced in a gene of interest. BCNK‐bearing polypeptides are further derivatized with a fluorophore (magenta diamond) via inverse‐electron‐demand Diels–Alder cycloaddition. (b) Proteolytic proteoform detection with genetic code expansion‐based molecular beacon. A fluorophore is introduced to a cellular protein as in (a), but its fluorescence is quenched by the presence of a matched fluorescence quencher (black diamond) attached to the same protein. Fluorescence signal is generated upon fluorophore‐quencher separation via proteolysis. (c) Genetic code expansion labeling of amyloid precursor protein (APP). HEK293 cells were transfected with transgenes encoding APP(TAG)‐myc mutants, PylRS‐AF and PylT and incubated with BCNK. Cells were then labeled live with tetrazine‐cy5, fixed and counterstained with anti‐myc for APP expression, and anti‐FLAG for PylRS‐AF expression. More fields of view are shown in Figure S2.

Here we report a composite labeling strategy that unites site‐specific probe placement capability of genetic code expansion with a turn‐on fluorescence ability upon proteolytic processing, to allow selective visualization of proteolytic proteoforms. The selective signal is elicited via a molecular beacon‐type design: the fluorescence signal on the desired proteolytic proteoform(s) is suppressed via Förster resonance energy transfer (FRET)‐based fluorescence quenching when the proteoform is still embedded in the protein precursor, but is activated when the proteoform is physically separated from the precursor via proteolytic processing (Figure 1 b). Precursor‐specific quenching is achieved via site‐specific attachment of a quencher to the portion of the precursor protein that will be cleaved from the desired proteolytic proteoform after processing. While a minimal tag (single amino acid) is required for minimally perturbative labeling of the proteoform of interest, the only constraint on tag size for the precursor or undesired proteolytic product to which the quencher will be appended is achieving optimal fluorophore‐quencher distance. Therefore, several protein labeling methods, such as SNAP,^[7]^ HaloTag,^[8]^ and PRIME,^[9]^ can be used to append the quencher to the precursor protein.

We demonstrate the molecular beacon‐style detection on amyloid‐β (Aβ): 36–43‐amino acid peptide species that are generated from sequential proteolytic processing steps of amyloid precursor protein (APP) by the β‐site APP cleaving enzyme (β‐secretase) followed by γ‐secretase.^[10]^ While Aβ peptides’ in vitro aggregation kinetics^[11]^ as well as processing enzymes involved in the generation of the peptides^[12]^ are relatively well understood, much less is known about the cellular dynamics of processing of APP to generate Aβ peptides, and their aggregation, due to the lack of tools to visualize natively processed Aβ in cells. Direct fusion of GFP to Aβ interferes with its oligomerization kinetics^[13]^ and cannot recapitulate the cellular environments in the endocytic and retrograde pathways‐where β‐ and γ‐secretases reside and can generate Aβ from APP^[14]^‐that likely influence Aβ properties in the cell. Understanding the spatiotemporal context in which Aβ is produced and accumulated may provide insight into pathogenicity of Aβ as well as therapeutic development targeted toward APP and Aβ for Alzheimer's disease.

To label natively processed Aβ, we selected several amino acid positions within the Aβ portion of APP695 (the predominant neuronal isoform of APP) that are not involved in post‐translational modifications (Figure S1a) to be replaced with a bicyclononyne‐lysine unnatural amino acid (BCNK). BCNK has extensively been used as an amino acid for protein labeling due to its efficient charging onto pyrrolysyl tRNA_CUA_ (PylT) via an engineered *Methanosarcina mazei* pyrrolysyl‐tRNA synthetase bearing Y306A/Y384F mutations^[15]^ (PylRS‐AF), stability in cells,^[4c]^ and excellent cycloaddition rates with tetrazines.^[4b]^ Three amber variants of APP‐H609TAG, H610TAG, and Q611TAG showed strong BCNK‐dependent expression (Figure S1b,c), and all produced two major bands corresponding to mature APP and immature, core‐glycosylated APP,^[16]^ with mature:immature APP ratios similar to that of wild‐type APP (Figure S1d), suggesting that BCNK incorporation into APP does not affect its maturation. Labeling of these APP(BCNK) variants with a membrane‐impermeable tetrazine‐cy5 (to confine labeling to APP that has trafficked to the cell surface) produced fluorescent rings at the cell membrane, consistent with specific labeling of APP (Figure 1 c and S2, quantifications in Figure S3), with minimal labeling background from translation readthrough of endogenous amber stop codons (Figure S4a,b). We chose the H609TAG variant‐with its excellent BCNK‐dependent APP production and BCNK incorporation site furthest from any proteolytic cleavage site‐to develop into an Aβ reporter.

To verify that Aβ can be processed out of APP(BCNK) and that fluorescent signals from APP vs. fully processed Aβ can be differentiated, we designed a doubly labeled fluorescent reporter in which the Aβ portion of APP is labeled with cy5 via genetic code expansion, and the C‐terminus of APP tagged with enhanced GFP (EGFP) (Figure 2 a). Co‐localized cy5 and EGFP signals correspond to full‐length or partially processed APP precursor, while standalone cy5 signals indicate fully processed Aβ. We confirmed that APP(H609TAG)‐EGFP showed BCNK‐dependent expression (Figure 2 b) and could be labeled with tetrazine‐cy5. To demonstrate the reporter can be used to visualize de novo generated Aβ in real time, we performed tetrazine‐cy5 labeling on APP(H609BCNK)‐EGFP at 4 °C to minimize endocytosis (the process of which will initiate APP processing), then allowed APP to be endocytosed and processed and Aβ produced over time at 37 °C. Immediately after tetrazine‐cy5 labeling at 4 °C, we observed cy5 signal exclusively at the cell surface, with strong co‐localization between cy5 and EGFP signals (Figure 2 c). After incubation at 37 °C for 2 hours, we observed formation of intracellular fluorescent puncta, consistent with APP internalization (Figure 2 c,d). Cy5 puncta are specific to transfected cells, suggesting they were labeled Aβ(BCNK)‐containing species, not non‐specific sticking of probes (Figure S4c). The cy5 labeling pattern also indicated we captured post‐endocytic APP and its derivatives, as the APP(H609BCNK)‐EGFP subpopulation residing at the ER envelope that has not yet trafficked to the cell surface is not labeled with cy5. The ability to precisely track post‐endocytic APP and its processing differentiated our reporter from other dual‐labeled APP reporter designs.^[17]^

**Figure 2:**
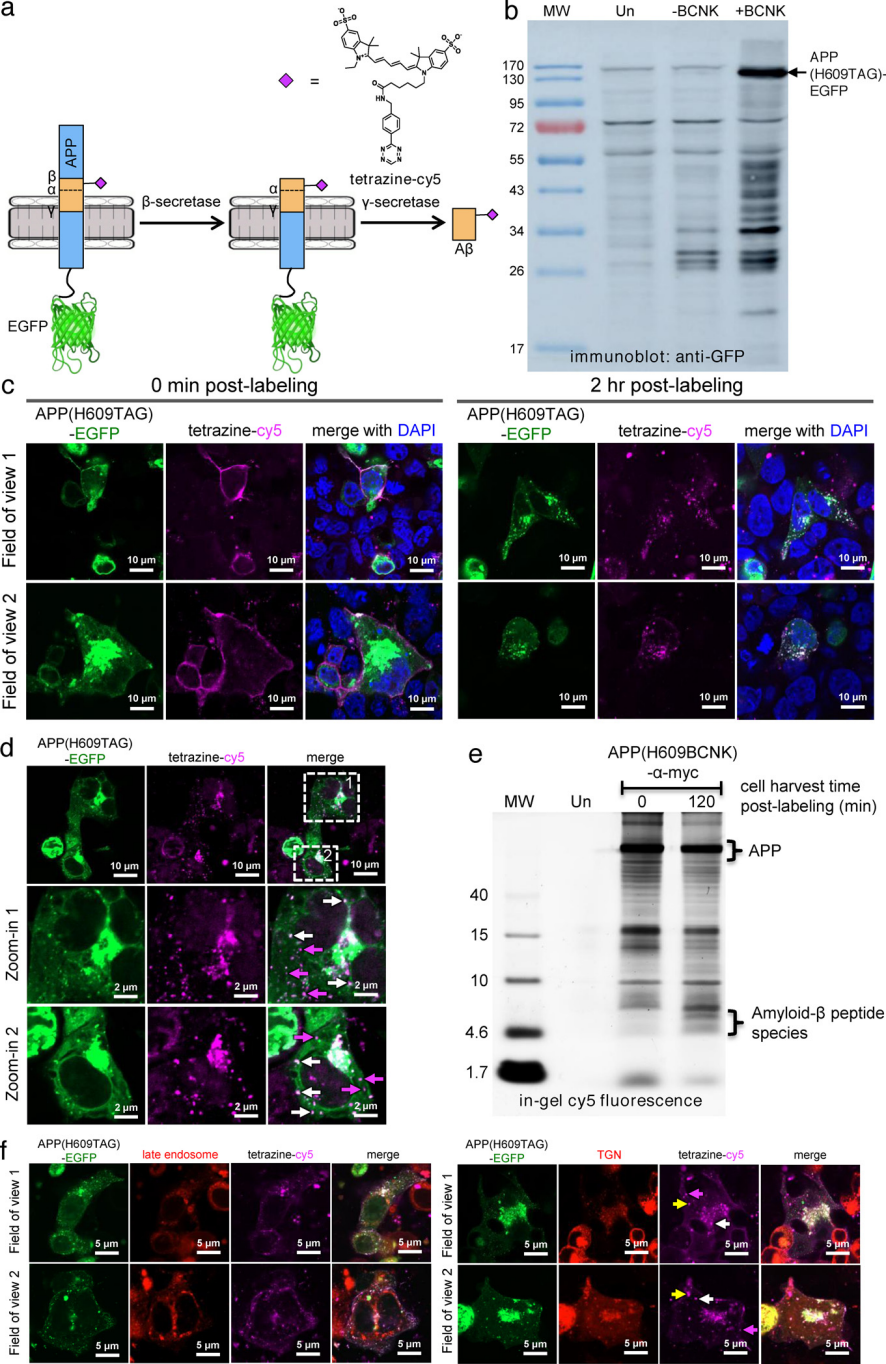
Verification of amyloid‐β (Aβ) peptide processing from APP. (a) Bifluorescent reporter design. BCNK is incorporated into the Aβ segment of amyloid precursor protein (APP) fused to EGFP via genetic code expansion, and subsequently derivatized with tetrazine‐cy5. Upon imaging, coincident EGFP and cy5 signals indicate full‐length or partially processed APP, while standalone cy5 signals represent fully processed Aβ. (b) APP(H609TAG)‐EGFP expression via genetic code expansion. HEK293 cells were transfected with transgenes encoding APP(H609TAG)‐EGFP, PylRS‐AF and Pyl‐tRNA_CUA_, and incubated with BCNK. Cell lysates were analyzed with western blot using anti‐GFP antibody. Un, untransfected cells. (c) Imaging post‐endocytic processing of APP and production of Aβ. HEK293 cells were transfected and incubated with BCNK as in (b). Cells were labeled live with tetrazine‐cy5 at 4 °C for 30 minutes and immediately fixed for imaging (0 min post‐labeling) or allowed to grow for additional 2 hours at 37 °C to permit APP internalization and processing before fixation (2 h post‐labeling). (d) Zoom‐ins of a 2 h post‐labeling cell sample prepared similarly as in (b). Some coincident EGFP and cy5 puncta are marked with white arrows. Standalone cy5 puncta are marked with magenta arrows. (e) Cy5‐labeled amyloid‐β species is generated from cy5‐labeled APP post‐endocytosis. HEK cells expressing APP(H609BCNK) were labeled as in (b) and either lyzed immediately, or incubated at 37 °C for 2 hours to allow APP processing before lysis. Protein content was separated on SDS‐PAGE gel and its cy5 fluorescence analyzed in‐gel. The bracket highlights low‐molecular weight species (between 4–10 kD ladder), consistent with the size of amyloid‐β peptides. Un, untransfected cells. (f) Localization of APP and Aβ to the trans‐golgi network (TGN). HEK293 cells were transfected with a plasmid expressing mApple‐TGN38 (TGN marker) or mApple‐Rab7a (late endosome marker), along with other genetic code expansion transgenes. Cells were labeled with tetrazine‐cy5 and incubated for 2 h post‐labeling at 37 °C. Yellow arrows indicate fully processed Aβ in TGN; magenta arrows indicate fully processed Aβ outside of TGN; white arrows indicate full‐length or partially processed APP in TGN.

While most cy5 puncta co‐localize with EGFP puncta (Figure 2 d, white arrows)‐indicating that these are intact or partially processed APP that still contains the Aβ segment‐we could observe EGFP‐only puncta which represent vesicles containing the C‐terminus of APP liberated after secretase cleavage, as well as cy5‐only puncta (Figure 2 d, magenta arrows) which represent fully processed amyloid‐β. We further confirmed via in‐gel cy5 fluorescence imaging the formation of cy5‐labeled peptide species ≈4kD in size, consistent with amyloid‐β, after APP internalization (Figure 2 e). These amyloid species can be stained with anti‐amyloid antibody (which also stained the Aβ segment of APP, Figure S5a,b), but cannot be counterstained with plaque‐specific Amylo‐Glo, suggesting they have not formed plaque ultrastructure (Figure S5c). In our in‐gel fluorescence experiment, we did not observe cy5‐labeled soluble amyloid precursor protein α (sAPPα, ≈100kD in size), which could be generated from non‐amyloidogenic processing of APP by α‐secretase either before or after APP internalization. This could be because α‐secretase processing occurs primarily at the secretory pathway and/or the cell surface where sAPPα can be secreted and diffuse away, and β‐secretase processing occurs much more efficiently in the endocytic pathway compared to α‐secretase processing.^[18]^ We note that the punctate Aβ localization pattern revealed by our reporter is distinctly different from diffuse staining observed if Aβ is produced exogenously from an expression plasmid (Figure S6). This highlights the need to study Aβ under the physiological conditions in which it is generated. Both the post‐endocytic pool of APP (coincident EGFP and cy5 signals) and fully processed Aβ (cy5 signals with no EGFP) primarily localized to the *trans*‐Golgi network (TGN, marked by TGN38), with no observable distribution to late endosomes (marked by Rab7a) (Figure 2 f). This is consistent with observations in which Aβ40, the predominant species of Aβ,^[19]^ is generated in TGN from surface populations of APP that have been endocytosed.^[20]^

After verification that Aβ can be processed from APP(BCNK), we proceeded to construct a molecular beacon reporter to detect intracellular Aβ. We replaced EGFP of APP(H609TAG)‐EGFP with HaloTag, and confirmed its BCNK‐dependent expression (Figure 3 a,b). HaloTag can be derivatized with QSY21, a non‐fluorescent acceptor with optimal absorbance for quenching of cy5 fluorescence.^[21]^ In this molecular beacon configuration, cy5 on the Aβ segment of APP should be quenched by an intramolecular HaloTag‐linked QSY21, and fluorescent only when Aβ is fully released from APP (Figure 3 a). Our molecular beacon design places the FRET donor cy5 on the extracellular side of APP and the FRET acceptor QSY21 on the cytosolic side. While such transmembrane FRET has rarely been investigated, the distance spanning the lipid bilayer (≈40 Å) is within the reported 40–60 Å Förster radius of cy5‐QSY21 energy transfer.^[21]^ FRET is also a through‐space interaction and should not be sensitive to steric or electrostatic occlusion.

**Figure 3:**
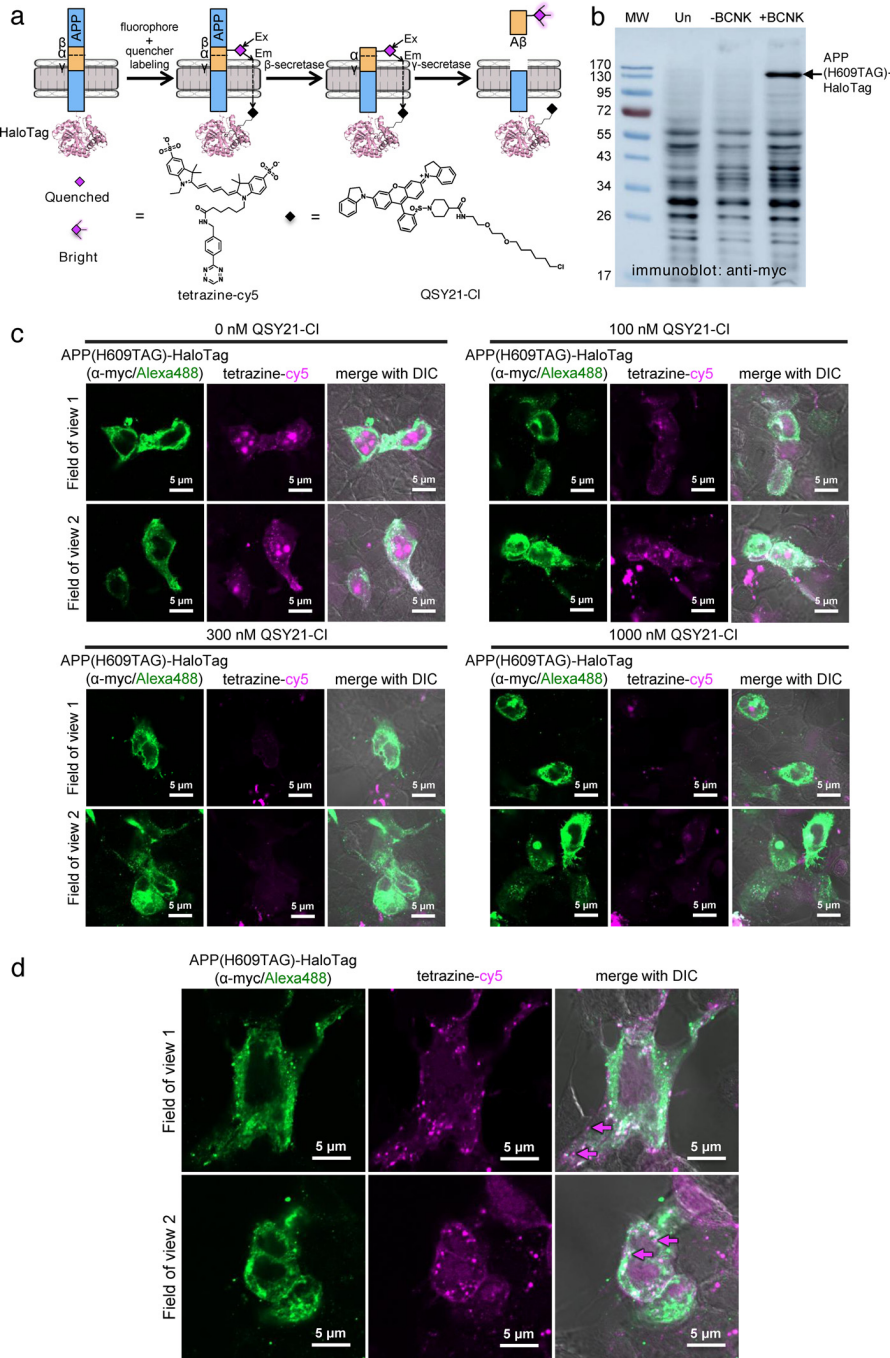
Genetic code expansion‐based molecular beacon to detect de novo generated Aβ peptides. (a) Molecular beacon reporter design. APP(H609BCNK)‐HaloTag is doubly labeled with tetrazine‐cy5 and QSY21‐chloroalkane (QSY21‐Cl). Cy5 fluorescence is turned on only when cy5‐containing Aβ is proteolytically processed from APP. (b) APP(H609TAG)‐HaloTag expression via genetic code expansion. HEK293 cells were transfected with transgenes encoding APP(H609TAG)‐HaloTag, PylRS‐AF and Pyl‐tRNA_CUA_, and incubated with BCNK. Cell lysates were analyzed with western blot using anti‐myc antibody. More western blot analyses with different antibodies are shown in Figure S7. (c) Dose‐dependent quenching of cy5 fluorescence on APP with QSY21‐Cl. HEK293 cells were transfected as in (b) and incubated with BCNK. Cells were labeled live with tetrazine‐cy5 and indicated concentrations of QSY21‐Cl at 4 °C for 30 minutes, immediately fixed, and APP‐HaloTag immunostained via its C‐terminal myc tag for imaging. (d) Zoom‐ins of cells prepared as in (b), which were further incubated for 2 hours at 37 °C post‐labeling to permit APP internalization and processing. Some fully processed Aβ are marked with magenta arrows.

We performed double labeling of APP(H609BCNK)‐HaloTag with tetrazine‐cy5 and QSY21 conjugated to a HaloTag chloroalkane ligand (QSY21‐Cl, characterizations in Figure S8), and tested dose‐dependent quenching of cy5 via increasing concentrations of QSY21‐Cl. We found that labeling with 300 nM QSY21‐Cl resulted in quenching of ≈79 % of cy5 fluorescence on APP, confirming that transmembrane FRET‐based quenching can occur efficiently (Figure 3 c, quantifications in Figure S9). Quenching of the cy5 signal by QSY21 does not occur when HaloTag is not fused to APP(H609BCNK), demonstrating a requirement for the proximity of the QSY21 quencher to the cy5 fluorophore within the same APP molecule (Figure S10). Further incubation of cells at 37 °C allowed post‐endocytic APP processing, resulting in markedly increased cy5 fluorescence, the punctate pattern of which is distinct from that of APP‐HaloTag (Figure 3 d). In contrast to cy5 puncta observed with APP(H609BCNK)‐EGFP which represented a mixture of the APP precursor and processed products, cy5 signal generated from the processed molecular beacon rarely overlapped with APP, suggesting that it represents *bona fide* fully liberated Aβ peptide. Puncta with coincident anti‐myc staining on APP and cy5 could still be observed with APP(H609BCNK)‐HaloTag(QSY21), which may indicate incomplete quenching‐a feature we will improve in future designs‐or molecularly separated Aβ and APP in the same vesicle that cannot be optically separated due to diffraction‐limited imaging.

In summary, by combining genetic code expansion labeling with a molecular beacon design, we have created a prototype reporter for amyloid precursor protein processing and intracellular amyloid‐β production. Such a molecular beacon Aβ reporter should be useful for facile monitoring of Aβ biogenesis in cells‐especially neurons‐and could be coupled to high‐content imaging platforms for screening of compounds which can modulate Aβ biogenesis. Further optimizations to the labeling system to minimize perturbation to Aβ biogenesis and property can be performed, for example via the use of even smaller amino acids and labels.^[22]^ Beyond amyloid‐β, we expect that this advance will enable further live‐cell imaging studies of other proteolytic proteoforms such as various neuropeptides and cytokines that cannot be studied with conventional tagging techniques.

## Conflict of interest

K.S., K. J., and C.U. have filed a patent related to the molecular beacon reporter design for amyloid peptides.

## Supporting information

As a service to our authors and readers, this journal provides supporting information supplied by the authors. Such materials are peer reviewed and may be re‐organized for online delivery, but are not copy‐edited or typeset. Technical support issues arising from supporting information (other than missing files) should be addressed to the authors.

Supplementary
